# A case of ductal carcinoma in situ (DCIS) with markedly elevated CA15-3 levels requiring 2 years of diagnosis

**DOI:** 10.1186/s40792-023-01792-8

**Published:** 2023-12-01

**Authors:** Mutsumi Fujimoto, Yoshie Kobayashi, Kazuya Kuraoka, Tomoyuki Yoshiyama, Hideo Shigematu

**Affiliations:** 1 Department of Breast Surgery, Kure Medical Center and Chugoku Cancer Center, 3-1 Aoyama-Cho, Kure, Hiroshima 737-0023 Japan; 2 Department of Diagnostic Pathology, Kure Medical Center and Chugoku Cancer Center, 3-1 Aoyama-Cho, Kure, Hiroshima 737-0023 Japan

**Keywords:** DCIS, CA15-3, Tumor marker

## Abstract

**Background:**

CA15-3 is often elevated in breast cancer recurrence and rarely in ductal carcinoma in situ (DCIS). We report a case of DCIS with elevated CA15-3 levels, which was diagnosed after over 2 years of follow-up.

**Case presentation:**

A 74-year-old woman presented with a left-sided breast mass and pain. Redness, swelling, and induration were observed in the left breast. Ultrasonography revealed a non-mass lesion in the left 3 o'clock position, skin thickening, and axillary lymphadenopathy. Serum CA15-3 levels were markedly high at 640 U/mL, suggesting inflammatory breast cancer. However, biopsies showed no malignancy. We diagnosed chronic mastitis with elevated CA15-3 levels and followed up with magnetic resonance imaging and a biopsy, as needed. Finally, DCIS was diagnosed 27 months after the first visit. She underwent a left mastectomy and a sentinel lymph node biopsy; DCIS had spread 6.5 cm and was immunohistochemically positive for CA15-3. No metastasis was found in the lymph nodes, but incidental Hodgkin lymphoma was observed. Postoperative normalization of CA15-3 levels indicated that she had DCIS with elevated CA15-3 levels. The patient underwent chemotherapy for Hodgkin lymphoma postoperatively, and there was no evidence of recurrence 1 year after surgery.

**Conclusion:**

High CA15-3 levels can also be observed in DCIS, indicating that CA15-3 should not be used solely in breast cancer staging.

## Background

CA15-3 is a tumor marker that is commonly used in breast cancer practice. It is often elevated in recurrent breast cancer and is measured for postoperative monitoring of early-stage breast cancer and disease assessment in advanced breast cancer [1]. CA15-3 levels may also be elevated in stage I–III breast cancer, but elevated CA15-3 levels are rarely found in ductal carcinoma in situ (DCIS). We report a case of DCIS with markedly elevated CA15-3 levels that required 2 years for diagnosis.

## Case presentation

**Fig. 1:**
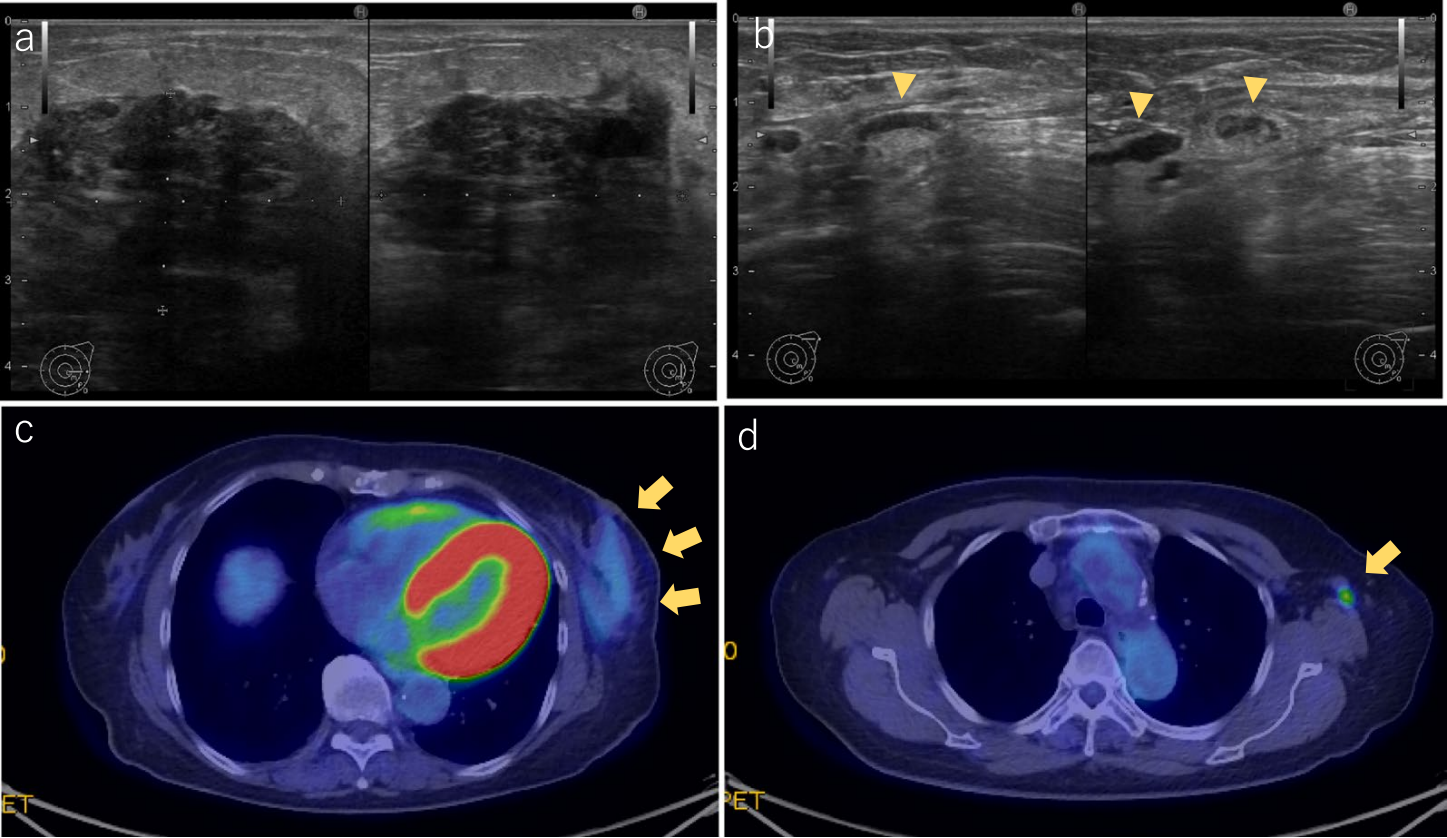
Clinical imaging findings at initial examination. **a** Ultrasound of the breast: a 3.8 × 2.5 × 3.5 cm large non-mass lesion from below the nipple to the 3 o'clock position. **b** Lymph nodes with cortical thickening in the left axilla (arrowhead). **c**, **d** PET-CT: SUVmax 2.1 in the left mammary gland and SUVmax 3.5 in the left axillary lymph node (arrow). PET-CT, positron emission tomography-computed tomography

A 74-year-old woman presented to the emergency department with a mass in her left breast and a two-month history of pain in the breast. The patient had a history of necrosis of the right femoral head. She was a non-smoker. There was no family history of breast or ovarian cancers. Physical examination revealed redness, swelling, and induration of the left breast. Ultrasonography of the breast revealed a non-mass lesion in the left 3 o'clock position, skin thickening of the left breast, and cortical thickening of the left axillary lymph nodes (Fig. 1a, b).

**Fig. 2:**
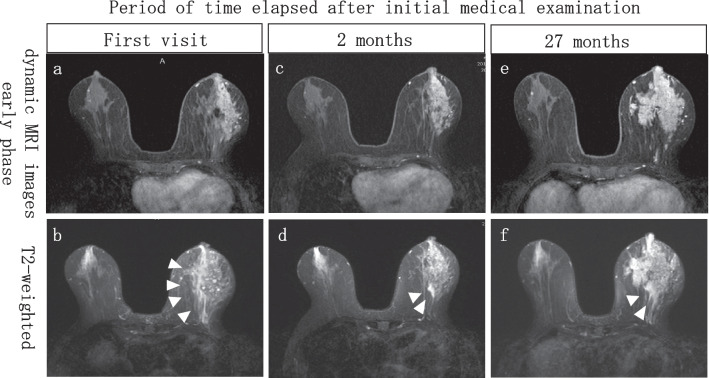
Changes in MRI findings. **a**, **b** At initial diagnosis, **c**, **d** 2 months after initial diagnosis, **e**, **f** at DCIS diagnosis. The high-signal intensity of the dorsal part of the mammary gland in the fat-suppressed T2-weighted image is shown with arrowheads. MRI, magnetic resonance imaging; DCIS, ductal carcinoma in situ

Dynamic breast magnetic resonance imaging (MRI) revealed extensive non-mass enhancement in the left 3 o'clock position and high signal intensity in the dorsal part of the mammary gland on fat-suppressed T2-weighted images (Fig. 2a, b). Blood tests showed CA15-3 levels at 640 U/mL and CEA at 25.9 ng/mL. Positron emission tomography-computed tomography (PET-CT) showed only mild enhancement in the left breast and left axillary lymph nodes, with no other abnormal enhancement (Fig. 1c, d). A 16-G core needle biopsy of a non-mass lesion in the left mammary gland showed ductal hyperplasia with intense lymphocytic infiltration of the stroma. A skin biopsy of the left breast showed edema of the dermis but no malignant findings. A needle biopsy of the left axillary lymph node showed an increased number of small-to-medium-sized lymphocytes, which, after further immunohistochemical examination, led to the diagnosis of reactive lymphoid hyperplasia. Based on the above findings, the patient was diagnosed with chronic mastitis with elevated CA15-3 levels and was referred for follow-up.

## Clinical progress

**Fig. 3:**
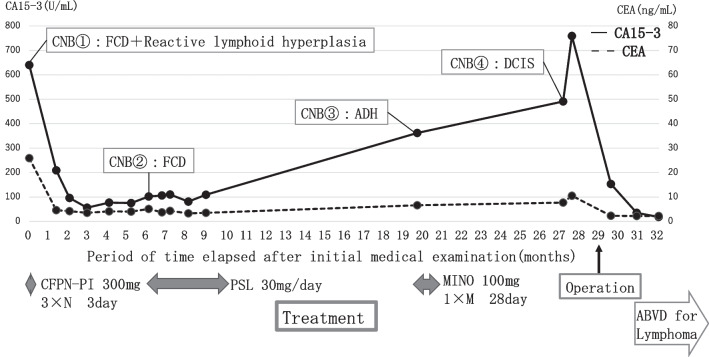
Time course of tumor markers from the first visit to the postoperative period. FCD: Fibrocystic disease, ADH: Atypical ductal hyperplasia, DCIS: Ductal carcinoma in situ, CFPN-PI: Cefcapene pivoxil, MINO: Minocycline, PSL: Prednisolone

She was treated with antibiotics (cefcapene pivoxil and minocycline) for chronic mastitis, but her symptoms remained unchanged. A dynamic breast MRI performed 2 months after the initial examination showed that the enhanced areas in the left breast remained unchanged. However, there was a reduction in the high signal intensity on fat-suppressed T2-weighted images in the dorsal side of the mammary gland, the pain subsided to mild pain, and there was a decrease in CA15-3 levels (Figs. 2c, d, 3). A contrast-enhanced breast MRI 6 months after the initial diagnosis showed enlarged enhancement areas. A second 16-G core needle biopsy was performed, but no malignant findings were found.

Considering the possibility of persistent inflammation, she was treated with prednisolone 30 mg/day, without any effects. The dose was gradually reduced and discontinued after 4 months. Further, twenty months after the initial diagnosis, a 16-G core needle biopsy was again performed because of induration of the left breast and an enlarged enhanced area on dynamic breast MRI, which showed partial atypical ductal hyperplasia of the breast but no malignant findings.

Then, twenty-seven months after the initial diagnosis, CA15-3 levels were elevated again, and dynamic breast MRI showed an enlarged enhancement area and high signal intensity on fat-suppressed T2-weighted images of the dorsal side of the breast (Figs. 2e, f, 3). Because an enlarged left breast mass was also observed on palpation, a fourth 16-G core needle biopsy was performed, and a low-grade DCIS was diagnosed.

## Operative management

**Fig. 4:**
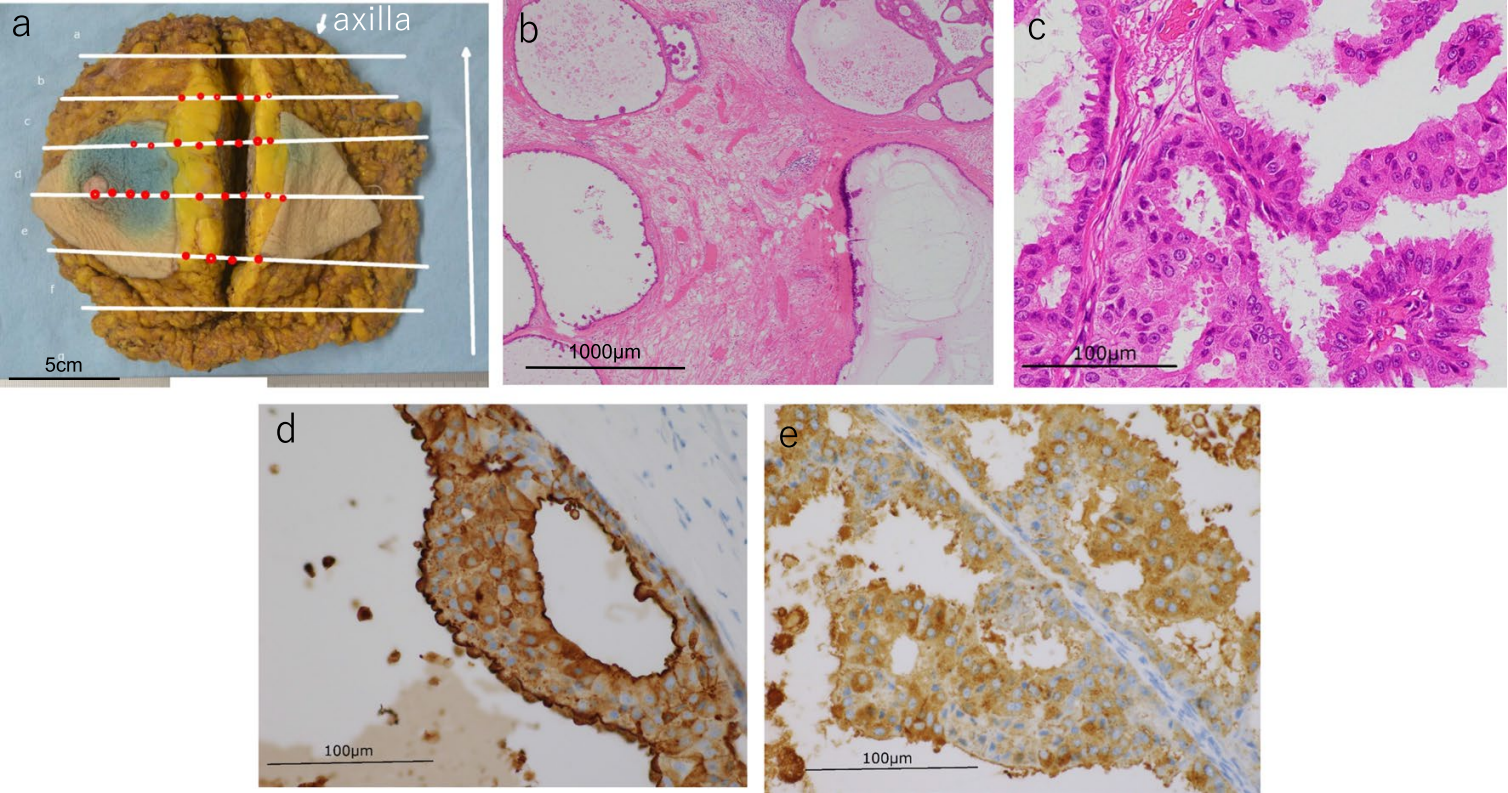
Pathological findings. **a** Mapping of surgical specimens. Red dots indicate the extent of DCIS. **b**, **c** HE staining showing low papillary type DCIS, with surrounding vasodilatation, stasis, and collagen fiber hyperplasia. **d** Expression of CA15-3. **e** Expression of CEA. CA15-3 and CEA are positive for plasma membrane and cytoplasm

Total left mastectomy and sentinel lymph node biopsy were performed. One enlarged axillary lymph node was removed in addition to the sentinel lymph nodes. The excised specimen showed high-grade DCIS over an area of 6.5 × 6 × 3.6 cm (Fig. 4a–c). Vasodilatation, stasis, and collagen hyperplasia are seen around DCIS. CA15-3 expression was examined via immunohistochemical staining with NCL-MUC-1 (Leica Biosystems, Wetzlar, Germany) and CEA with NCL-L-CEA-2 (Leica Biosystems). Immunohistochemical staining was performed using the Ventana BenchMark ULTRA (Roche Diagnostics, Basel, Switzerland). Breast cancer cells were immunohistochemically positive for CA15-3 and CEA in the plasma membrane and cytoplasm (Fig. 4d, e). There were no breast cancer metastases in the sentinel nodes. However, there was multifocal hyperplasia of large lymphocytes in an enlarged lymph node, and the immunohistochemical results led to the diagnosis of classical Hodgkin lymphoma.

Based on the above findings, the patient was diagnosed with DCIS in the left breast, complicated by Hodgkin lymphoma. The CA15-3 levels normalized quickly after surgery. One year postoperatively, there was no breast cancer recurrence or CA15-3 re-elevation. Classical Hodgkin lymphoma was treated with six courses of ABVD (Doxorubicin, Bleomycin, Vinblastine, Dacarbazine) and remitted without relapse.

## Discussion

CA15-3 is associated with breast cancer stage and disease status [2]. Elevated CA15-3 levels are rare in early breast cancer, occurring in less than 10% of stage I cases [1, 3]. Furthermore, elevated CA15-3 levels are extremely rare in patients with DCIS. In Japan, one case of DCIS with elevated tumor markers, CA15-3, NCC-ST439, and BCA255 [4], one case with elevated CA15-3 and NCC-ST439 [5], and two cases with elevated CEA levels [6, 7] have been previously reported. Extensive intraductal involvement was a common feature between these reports and the present case (Table 1). We considered the mechanisms by which CA15-3 is elevated in DCIS.

**Table 1:** Reports of DCIS with elevated tumor markers in Japan

Reference	Age	Subtype	DCIS width	CA15-3	CEA	Others
Tani et al. [6]	73	DCIS	No data (extensive)	No data (normal)	17	Postoperative colon cancer
Terasawa et al. [7]	56	NE-DCIS	7.0 cm	No data (normal)	7.09	Postoperative ovarian cancer
Yamamuro et al. [4]	40	Papillary type	9.0 cm	103.5	No data (normal)	NCC-ST439: 578.4 U/mL BCA255: 291.7 U/mL
Ichihara et al. [5]	43	Micropapillary carcinoma in situ	4.0 cm	198.5	No data	NCC-ST439: 1400 U/mL
Present case	74	Low papillary type	6.5 cm	491	10.5	Complicated Classical Hodgkin's Lymphoma

DCIS: ductal carcinoma in situ; NE-DCIS: neuroendocrine carcinoma in situ

First, CA15-3 is expressed only on the luminal surface of normal breast tissue. However, in chronic inflammatory conditions, CA15-3 loses polarity and is expressed throughout the plasma membrane [8]. Meanwhile, in breast cancer cells, it is also expressed in the cytoplasm [9]. Mommers et al. reported that 22% of highly differentiated DCIS and 46% of poorly differentiated DCIS also showed CA15-3 expression on the basement membrane side [9].

**Fig. 5:**
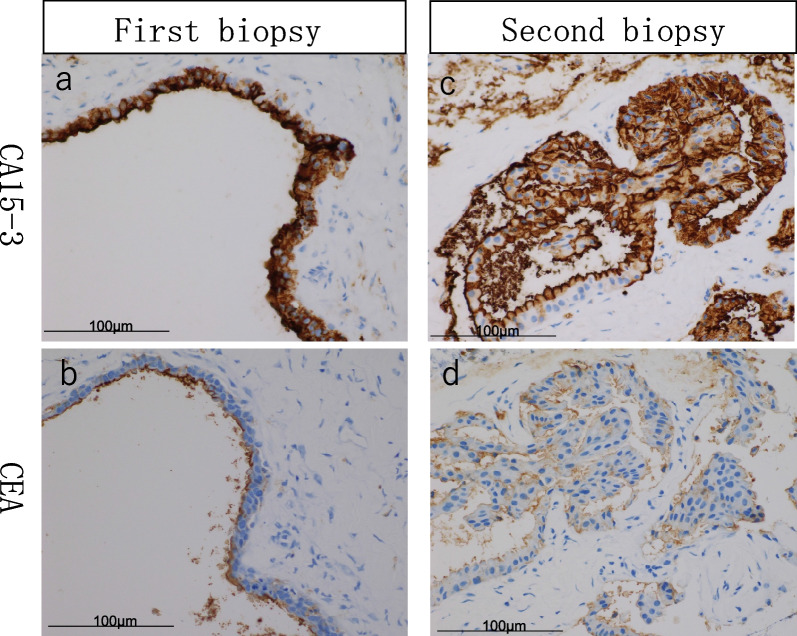
Expression of CA15-3 and CEA in core needle biopsy. **a** Expression of CA15-3 and **b** CEA in the first biopsy. CA15-3 was strongly positive throughout the mammary gland cells, and CEA was expressed only on the luminal surface. **c** Expression of CA15-3 and **d** CEA in the second biopsy. After the tumor markers had spontaneously decreased, CA15-3 was expressed on the luminal surface

In the present case, CA15-3 was also positive in the plasma membrane and cytoplasm of the breast cancer cells, leading to a change in polarity. To elucidate the mechanism of CA15-3 elevation, we retrospectively performed an additional immunohistochemical evaluation of tissues at initial presentation and during follow-up (Fig. 5). The results showed a high CA15-3 expression at the initial examination. However, at the second needle biopsy, when tumor markers had decreased, CA15-3 expression had decreased, and normal polarity was observed. Retrospective immunostaining showed that serum CA15-3 was altered along with changes in CA15-3 polarity. The tissue exhibited high levels of CA15-3 expression, but elevated CA15-3 expression alone is not a conclusive diagnostic indicator of cancer. Biopsies were performed at different sites and times because malignancy was suspected, but no malignant findings were confirmed. This case, while uncommon, was diagnosed as a benign disease associated with elevated levels of CA15-3 and was subject to regular follow-up. In addition to testing for CA15-3, we also evaluated the expression of CEA in specimens from the first and second needle biopsies; CEA was localized to the luminal surface in both biopsies (Fig. 5). Although the levels of CA15-3 and CEA in serum showed similar trends, there is no satisfactory explanation for the differences in the expression patterns of the two markers between these tissues. However, it is suggested that this may be due to tumor heterogeneity.

Second, inflammation of the breast tissue has also been implicated. In this case, pain and redness of the breast were observed at the time of the initial examination, and histological biopsy showed infiltration of stromal inflammatory cells. In addition, fat-suppressed T2-weighted breast MRI showed high signal intensity in the dorsal side of the mammary gland, a finding that reflects lymphocytic infiltration, vasodilation, and increased vascular permeability [10, 11].

The clinical course of the patient showed improved subjective symptoms and a decrease in CA15-3 levels, with a decrease in high signal intensity on fat-suppressed T2-weighted MRI. However, preoperative examination showed increased high signal intensity in the dorsal side of the mammary gland and re-elevation of CA15-3. These findings suggested that inflammation of breast tissue may be involved in the migration of CA15-3 into the blood. However, treatment with antibiotics and steroids was unsuccessful, and the cause of inflammation remains unknown. This needs to be investigated in future case series.

In addition, there were no reports of elevated CA15-3 levels in lymphoma, although classical Hodgkin lymphoma was observed incidentally. Further, the fact that tumor markers improved in the early postoperative period and before the treatment for lymphoma led us to conclude that the cause of the elevated CA15-3 level was DCIS. In this case, inflammatory breast cancer was suspected at the initial diagnosis, but no malignant findings were found on the biopsy. Increased CA15-3 expression in peripheral blood may also be observed in carcinomas other than breast cancer, benign breast tumors, ovarian cancer, endometriosis, and ovarian cysts [12, 13]. In the present case, a systemic examination was performed after suspicion of other diseases, but no other lesions were found, and the patient was diagnosed with mastitis.

Follow-up with a diagnosis of chronic mastitis with an elevated CA15-3 level and continued frequent follow-up led us to the diagnosis of DCIS and early treatment. Our experience with this case suggests that immunohistochemical staining of tissue is useful to identify the lesion responsible for the elevated tumor marker, even in benign lesions. Further, it is important to continue regular surveillance of benign lesions with elevated tumor markers.

## Conclusion

We report a case in which CA15-3 was markedly elevated, but no malignancy was diagnosed, and DCIS was diagnosed after more than 2 years of follow-up. This case demonstrates that elevated CA15-3 levels may be present in benign disease and DCIS and that breast cancer staging based on CA15-3 may not be ideal.

## Data Availability

All data generated or analyzed during this study are included in this published article.

## Abbreviations

DCIS: Ductal carcinoma in situ
MRI: Magnetic resonance imaging
PET-CT: Positron emission tomography-computed tomography
FCD: Fibrocystic disease
ADH: Atypical ductal hyperplasia
CFPN-PI: Cefcapene pivoxil
MINO: Minocycline
PSL: Prednisolone
ABVD: Doxorubicin, Bleomycin, Vinblastine, Dacarbazine
